# Prophylactic Surgery in the BRCA+ Patient: Do Women Develop Breast Cancer While Waiting?

**DOI:** 10.3390/curroncol28010069

**Published:** 2021-01-25

**Authors:** Sheina A. Macadam, Karen Slater, Rona E. Cheifetz, Leigh Jansen, Stephen Chia, Penelope M. A. Brasher, Esta S. Bovill

**Affiliations:** 1 Division of Plastic & Reconstructive Surgery, Department of Surgery, University of British Columbia, Vancouver, BC V5Z 1M9, Canada; karennslater@gmail.com (K.S.); leigh.jansen@gmail.com (L.J.); esta@drbovill.com (E.S.B.); 2 Department of Surgery, University of British Columbia, Vancouver, BC V5Z 1M9, Canada; rcheifetz@bccancer.bc.ca; 3 Department of Medical Oncology, University of British Columbia, Vancouver, BC V5Z 1M9, Canada; schia@bccancer.bc.ca; 4 Center for Clinical Epidemiology and Evaluation, Vancouver Coastal Health Research Institute, Vancouver, BC V5Z 1M9, Canada; penny.brasher@vch.ca

**Keywords:** BRCA, breast cancer, breast reconstruction, prophylactic surgery

## Abstract

Breast cancer susceptibility gene (BRCA) mutation carriers have an increased risk of breast cancer. Mitigation of this risk can be achieved via surveillance or prophylactic mastectomy with or without breast reconstruction. Those that choose surgery expect to reduce their chance of developing cancer. The purpose of this study was to determine the incidence of patients developing breast cancer prior to surgery and to identify modifiable contributing factors within the patient journey. This is a historical cohort study of all BRCA mutation carriers identified through the British Columbia Cancer Hereditary Cancer Program between 2000 and 2012. Patients were divided into two groups: surveillance (S) and prophylactic mastectomy with immediate breast reconstruction (PM/IBR). The incidence of cancer, time to PM/IBR and patient journeys were analyzed. A total of 333 women were identified. The time to surgery from mutation disclosure was a median of 31 (5.3, 75.7) months. During this period, 6% of patients developed breast cancer compared with a 14% incidence of breast cancer in patients choosing surveillance. The majority of time to surgery was attributed to the period between mutation disclosure and the decision to proceed with surgery. Strategies to facilitate decision-making as well as wait list prioritization and dedicated operative time should be targeted to this population to decrease the number of women developing an interval cancer prior to surgery.

## 1. Introduction

The Breast Cancer Susceptibility Gene 1 (BRCA1) and the Breast Cancer Susceptibility Gene 2 (BRCA 2) are tumor suppressor genes that encode proteins involved in the repair of DNA double-strand breaks by way of the homologous recombination repair pathway [1]. Inherited mutations confer a heightened lifetime risk of breast cancer and/or ovarian cancer [2,3]. The detection of these mutations is important in the prevention and surveillance of the disease and since their discovery over 20 years ago there has been increased availability of genetic testing.

Once given a positive BRCA mutation result, individuals can begin surveillance that combines mammography and magnetic resonance imaging (MRI), clinician breast examination and breast self-examination commencing at age 25 to 35 years [4,5]. While heightened surveillance cannot prevent the development of breast cancer, it increases the likelihood of early detection. However, the sensitivity of mammographic surveillance is lower amongst younger women, which is problematic for BRCA mutation carriers in whom the risk for early onset (<40 years) breast cancer is increased up to 20-fold [6]. It has been estimated by Meijers-Heijboer et al. that 25% of young, high-risk women that undergo close surveillance will develop breast cancer and eventually die from the disease [7]. Even with a combination of MRI and mammography, the largest prospective single-center study to date has demonstrated a ten-year breast cancer-free survival of 74% for BRCA mutation carriers choosing surveillance [8]. Moreover, there are concerns that women may not comply with the schedule of regular examinations or that the radiation incurred by mammography may increase the risk of breast cancer in BRCA mutation carriers, who by the nature of the mutations have defective DNA repair mechanisms [9]. Given these factors, it is not surprising that many patients will decide to proceed with a risk-reducing prophylactic mastectomy with or without immediate breast reconstruction (PM/IBR) [10].

The process leading to a prophylactic mastectomy is complex. Patients require access to health services required to make an informed decision, consultation with specialized health care professionals, time to consider the physical and emotional consequences of this type of surgery and balancing this intervention with life events such as child-bearing. Therefore, once a woman receives a positive mutation result, the time to risk-reducing surgery is affected by a multitude of factors [11]. We examined the patient journey in those electing to proceed with risk-reducing surgery in order to determine the incidence of breast cancer prior to surgery and to compare this with patients choosing surveillance. We identified key milestones and modifiable decision points that might shorten the time to surgery and decrease the risk of development of cancer prior to prophylactic intervention.

## 2. Experimental Section

### 2.1. British Columbia (BC) Cancer Hereditary Cancer Program (HCP)

The HCP offers risk assessment, counseling and genetic testing. Those who test positive for BRCA1/2 are advised of options including surveillance, chemoprevention and prophylactic surgery. Patients can choose to be followed at the High Risk Clinic (HRC) where breast imaging and surgical referrals are arranged. The HRC maintains a database that includes demographics, genetic test results, a history of cancer diagnoses and a history of hereditary cancer-related surgery.

### 2.2. Patient Selection

A historical cohort study design was employed. The HRC database was interrogated to identify individuals with a positive BRCA1/2 mutation between 1 January 2000 and 31 December 2012. Patient charts were reviewed until 2018. Men, women younger than 25 years and patients who had any cancer diagnosis prior to genetic testing were excluded. Patients were divided into three groups based on a decision at the time of counseling: surveillance (S), prophylactic mastectomy with immediate breast reconstruction (PM/IBR) and prophylactic mastectomy only (PM).

### 2.3. Frequency of Surveillance and Surgical Techniques

Evidence based recommendations during the study period included a yearly breast MRI from age 25 to 65 and a yearly mammography from age 30, alternating every six months. The risk-reduction measures included a bilateral PM, a bilateral salpingo-oophorectomy (BSO) by age 40 and the use of risk-reducing medications [12]. Prophylactic mastectomies were either total, skin sparing or nipple-sparing. Patients that chose PM/IBR continued regular surveillance while awaiting surgery.

### 2.4. Definition of Time Periods

Time periods were defined as T1: BRCA mutation disclosure to decision for surgery; T2: decision to surgical consultation; T3: consultation to surgery.

### 2.5. Ethics

Approval was obtained from the University of British Columbia Clinical Research Ethics Board (CREB) and the Hereditary Cancer Program (HCP) within the British Columbia Cancer Agency (BCCA). The CREB waived the requirement for informed consent.

### 2.6. Statistical Analysis

The baseline patient characteristics for each patient group were described. Discrete variables were summarized by frequencies and percentages. Continuous variables were summarized by mean (SD) and median with percentiles (10th, 90th). Wait times were summarized with median with percentiles (10th, 90th). For the PM/IBR group time to surgery was summarized with a cumulative frequency curve. The cumulative incidence of breast cancer over time was calculated for each group; death was treated as a competing risk in all groups. A competing risks regression model was used to examine the differences in the development of cancer between the groups (Fine and Gray, JASA 1999) with group (S vs. PM/IBR), BRCA1 vs. BRCA2, years of hormone exposure and age included as covariates. A two-sided P value of less than 0.05 was considered to indicate statistical significance. All analyses were performed with the use of SAS 9.4 (SAS Institute Inc., Cary, NC, USA) and R version 3.4.1.

### 2.7. Funding Support

This study was supported by a grant from the Canadian Breast Cancer Research Alliance. This sponsor was not involved in the study design, data collection, analysis, interpretation of data or writing of the manuscript.

## 3. Results

### 3.1. Patient Demographics

A total of 1168 mutation positive patients were identified from the HCP database. Of those, 615 had a pre-existing cancer, 128 were men, 45 were younger than age 25 years, 44 had no patient records, one had had a previous PM and two were found to have no genetic mutation after a chart review. After exclusions 340 women were included. Of those, 201 patients chose S, 132 chose PM/IBR and seven chose PM. The PM group was excluded due to the small number of patients. The demographic characteristics and risk factor profile for each patient group are shown in Table 1. The median (10th, 90th percentiles) age of ascertainment of the BRCA1 or BRCA2 genetic mutation was 43 years (28, 66) in the S group and 38 years (29, 54) in the PM/IBR group. The BRCA1 mutation was more frequent in the PM/IBR group. Hormone exposure (defined as the time from the median population age of menarche to oophorectomy, cancer diagnosis or last follow-up) differed between the groups and was shorter in the PM/IBR group. Both groups were predominantly Caucasian.

### 3.2. Incidence of Breast Cancer

**Figure 1:**
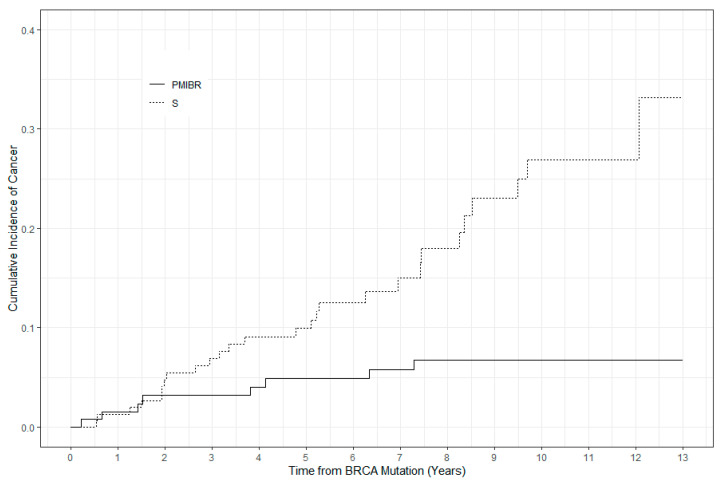
Mutation carriers choosing prophylactic mastectomy with immediate breast reconstruction vs. BRCA1/2 mutation carriers choosing surveillance.

Twenty-eight of the 201 surveillance patients and eight of 132 patients in the PM/IBR group developed invasive breast cancer during the study time period (Table 2; Table 3). The majority of these patients were compliant with screening (defined as imaging completed within 12 months or less). At five years, the cumulative incidence of breast cancer in the surveillance group was 9.9% compared with 4.9% in the PM/IBR group (difference = 5.0% (95%CI: 0–11.5%)) (Figure 1). After adjusting for prognostic factors, competing risks regression modeling showed a significantly higher risk of breast cancer in the S compared with the PM/IBR group (HR = 3.6 (95% CI: 1.4–9.1)).

### 3.3. Breast Cancer Characteristics

In the PM/IBR group six cases had an invasive ductal carcinoma (IDC) and two had a ductal carcinoma in situ (DCIS). Two of the eight cases were node positive, two were triple negative, three were HER2 positive, all were Grade 2 or higher and two were 2 cm or greater in size. In the S group 24 cases had an IDC and four had a DCIS. Five of 28 were node positive, seven were triple negative, five were HER2 positive and three were 2 cm or greater in size (Table 2 and Table 3).

### 3.4. Time to Surgery

**Figure 2:**
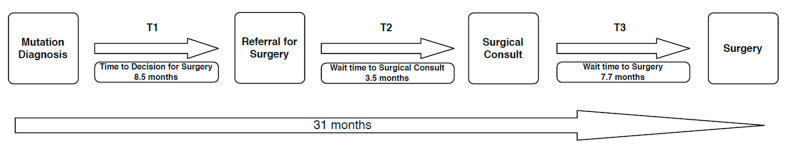
Mutation carriers choosing prophylactic mastectomy with immediate breast reconstruction.

The median time from mutation diagnosis to surgery in the PM/IBR group was 31.1 (5.3, 75.7) months. Patients waited 8.5 (1.9, 74.3) months from a BRCA mutation diagnosis to a decision for surgery (T1) with a 3.5 (1.1, 14.4) month time from a decision/referral to a surgical consultation (T2) and a 7.7 (1.5, 26.6) month time from a consult to surgery (T3) (Figure 2).

### 3.5. Reasons for Wait Time

For patients with available data, we compiled reasons for possible delays for proceeding with prophylactic surgical intervention. During the T1 time period we found 40% of patients made the decision to proceed within three months of disclosure of the BRCA mutation. Of the remaining patients, the majority initially declined a surgical consultation and then changed their mind at a later date for unknown reasons or reasons such as family influence, proceeding with child-bearing or proceeding with other surgery (i.e., a bilateral salpingo-oophorectomy). Other less frequent factors within the T1 time period included the long distance from a tertiary care center and a delay due to health issues. A total of 70% of patients went on to have their surgical consultation within six months. Of the remainder reasons for longer waits to consultation included a change in the requested surgeon, a long wait list for consultation or patient factors such as cancellation or a delay due to health issues. A total of 62% of patients had surgery within one year of their surgical consultation. Of the remainder factors leading to longer times included long surgical wait lists, the patient being undecided after consultation, the patient requesting a second opinion and patient health issues (Table 4).

Of the eight patients that developed cancer while awaiting PM/IBR surgery, six required more than three months to make the decision to proceed with surgery. The majority of these patients initially declined surgical consultation for an unknown reason and then changed their minds. One patient delayed for child-bearing and one patient changed her mind after being provided with new information (they initially thought imaging would prevent cancer).

### 3.6. Time to Development of Cancer

The median time from a BRCA diagnosis to cancer detection was 32.0 (2.7, 87.5) months in the PM/IBR group and 59.3 (14.9, 116.4) months in the S group. In the PM/IBR group five cancers occurred while awaiting surgery and three were found at the time of a mastectomy (Table 5).

## 4. Discussion

The goal of this study was to determine the incidence of BRCA mutation positive patients developing breast cancer prior to prophylactic surgery as well as modifiable patient and/or system factors that may decrease this incidence. We found a 31-month patient journey between BRCA mutation diagnosis disclosure and surgery with 6% of patients developing breast cancer during this time. This can be compared with the 14% incidence of breast cancer in patients choosing no intervention (surveillance), which is comparable with that found in other studies [13,14,15,16,17,18,19,20]. The mean time to cancer detection in the surveillance group was 59.3 months. The shorter time to diagnosis in the PM/IBR group may be due to the fact that a subset of the cancers was detected at the time of surgical intervention; these cancers may have gone undetected for longer in the surveillance group. We hypothesized that patients would face delays to consultation with specialized health care professionals and delays to surgery due to the long wait times for non-urgent medical consultations and surgery in Canada. However, we found that the majority of the time between mutation disclosure and surgery in the PM/IBR group was attributable to time to make the decision to proceed with surgery. This held true for the subset of patients that developed cancer prior to surgery.

The protective effect of a prophylactic mastectomy against the subsequent development of cancer in BRCA mutation carriers is well documented with the majority of studies showing zero incidence of cancer in patients after they have proceeded with a prophylactic mastectomy [13,14,15,16,17,18]. The development of cancer in patients after they have decided to proceed but prior to a prophylactic mastectomy is less studied and even fewer papers comment on the time to a prophylactic mastectomy. Ingham et al. reported five cases of occult cancer (3.9%) in 126 patients undergoing prophylactic surgery [15]. Kaas et al. reported one case of an occult IDC (5 mm in size) and six cases of an occult DCIS (4.7%) in 147 similar cases [16]. Heemskerk-Gerretsen et al. reported two cases of an occult DCIS and four cases of an occult IDC in 212 asymptomatic carriers at the time of a prophylactic mastectomy (2.8%) [18]. Skytte et al. reported one case of an IDC in 96 women undergoing a prophylactic mastectomy (1%) [14]. Yao et al. found a 2.7% incidental risk of an IDC at the time of a mastectomy in 150 BRCA mutation patients undergoing a prophylactic nipple-sparing mastectomy [21]. Of these studies only Skytte et al. in Denmark reported the time between BRCA mutation disclosure and a prophylactic mastectomy (11.5 months). Two additional studies comment on this time; Beattie et al. at the University of San Francisco reported a four month median time between BRCA mutation disclosure and risk-reducing surgery [22] and Flippo-Morton et al. in North Carolina reported a seven and a half month time [23]. Our study revealed a 6% incidence of breast cancer diagnosed prior to surgery or at the time of a prophylactic mastectomy. This is slightly higher than the cited studies and associated with a longer time period between BRCA disclosure and surgery (31 months).

The time to the decision to proceed with risk-reducing surgery (T1) is a key modifiable factor. This time period is complex as decision-making in the BRCA1/2 patient may be impaired due to psychological distress after the disclosure of the genetic mutation and the time required to make such an important decision [24]. Additionally, many patients may defer surgery for reasons such as a desire for child-bearing or may opt to start with surveillance and then subsequently decide to proceed with surgery, as was seen in our cohort. The genetic counselor is the first point of contact and it is incumbent upon this specialist to provide the time and information necessary for the patient to make an informed decision. Metcalfe et al. found that 28% of BRCA1/2 patients felt that they were not given sufficient information pertaining to prophylactic surgery at the time of the disclosure of their genetic mutation [25]. These authors went on to create a decision aid (DA) to help reduce decisional conflict. In a randomized controlled trial, BRCA1/2 patients were randomized to DA + standard care or standard care. The DA was a 15-page self-administered booklet summarizing information regarding the risk of breast cancer, preventive options, studies evaluating preventive strategies, guidelines for evaluating the evidence, risks and benefits associated with each option and definitions [26]. For a prophylactic mastectomy, the proportion of women undecided decreased by 11.5% in the intervention group and by 5.3% in the control group. Additionally, among women who were undecided about surgery, the mean cancer-related distress scores were significantly lower at 12 months for those in the intervention group than for those in the control group. Other studies have confirmed that decision aids are effective in reducing decisional conflict, the reduction of which would likely reduce T1 [27].

T2 and T3 are system-related and reflect the prioritization of more “urgent” patients within the health care system. BRCA mutation patients fall into a category somewhere between elective and urgent but are typically wait listed as elective while patients with active cancer are prioritized. Access to specialist consultation and the supply of elective surgery depends upon system capacity and the productivity with which that capacity is used [28]. Many studies have shown that productivity is affected by physician compensation with fee-for-service payment schedules increasing productivity. Other factors such as health expenditure per capita, hospital personnel and the number of surgical beds have an impact on wait times but the exact nature of these relationships is unclear and varied. In the case of the BRCA mutation patient at risk of developing cancer prior to prophylactic surgery, two main approaches can be considered. First, these patients need to be clearly prioritized on a surgeon wait list for consultation and surgery with a guaranteed maximum wait program for the delivery of service. Second, a dedicated block of operative time should be allocated to BRCA mutation patients in order to provide a rapid access model of care. This would ideally include two concurrently running operative rooms with surgical oncology and plastic surgery moving between rooms to perform the maximum number of surgical cases each operative day.

Despite the strengths of our study including a large sample size from a single institution and a sufficiently long follow-up period for breast cancer risk estimates, a prospective design would more accurately capture the exact reasons for the length of time between BRCA mutation diagnosis and prophylactic surgery. This would also allow the allocation of each cancer diagnosis into a specific time period (T1–T3) to further establish where to target resources. However, we have shown that at the 31-month time point from a BRCA mutation disclosure, 6% of BRCA patients awaiting prophylactic surgery (and still under surveillance) developed cancer. The majority of the patients that chose to proceed with PM/IBR and still developed cancer had a long T1 period, which may be attributed to a difficulty making the decision to proceed with risk-reducing surgery. This can be extrapolated to all healthcare systems that use similar hereditary cancer programs and be used to inform patients of the risk of developing cancer even after deciding to proceed with risk-reducing surgery. This may in turn focus centers on methods to optimize the decision-making process (T1) and, once a patient has decided to proceed, to ensure surgery is expedited in this high-risk group.

## 5. Summary

In conclusion, our data show that our cohort of BRCA mutation carriers that chose PM/IBR had a 31-month journey from mutation diagnosis to surgery and 6% were diagnosed with a DCIS or an IDC prior to or at the time of surgery. In the patients that developed an IDC, 25% had a node positive disease with 25% of tumors being greater than 2 cm in size. Women may therefore develop a high stage disease despite choosing to reduce their risk of cancer by undergoing prophylactic surgery. The implication is that if these patients had access to risk-reducing surgery earlier, the risk to life expectancy may have been altered. Strategies to decrease this wait time and thus decrease the incidence of cancer prior to surgery include the facilitation of decision-making as well as wait list prioritization. Dedicated operative time should also be targeted to this patient population in an effort to decrease the incidence and progression of occult disease.

## Figures and Tables

**Table 1:** Patient Characteristics.

Variable	Surveillance *n* = 201	Prophylactic Mastectomy/ Immediate Reconstruction *n* = 132
Age at BRCA diagnosis		
Mean (SD)—yr	43 (14.1)	40 (9.6)
Median—yr	43	38
10th, 90th Percentile	28, 66	29, 54
Length of follow-up after BRCA diagnosis		
Mean (SD)—yr	6.8 (4.2)	3.4 (2.7)
Median—yr	5.9	2.7
10th, 90th Percentile	1.6, 12.7	0.6, 7.2
BMI		
Mean (SD)—kg/m^2^	26 (6.7)	26 (5.6)
Median—kg/m^2^	24	24
10th, 90th Percentile	20, 35	20, 33
Ovarian hormone exposure		
Mean (SD)—yr	33 (13.8)	29 (9.3)
Median—yr	33	27.5
10th, 90th Percentile	16, 52	18, 41
Mutation—no. (%)		
BRCA1	87 (43.3)	78 (59.1)
BRCA2	112 (55.7)	53 (40.2)
BRCA1&2	1 (0.5)	1 (0.8)
Ethnicity—no. (%)		
Caucasian	142 (70.6)	107 (81.1)
Asian	6 (3)	2 (1.5)
South Asian	14 (7)	5 (3.8)
Hispanic	0 (0)	0 (0)
White Hispanic	0 (0)	0 (0)
Aboriginal	4 (2)	5 (3.8)
Multiple	2 (1)	1 (0.8)
Unknown	33 (16.4)	12 (9)

SD: standard deviation; BMI: body mass index.

**Table 2:** Cancer Characteristics: Surveillance Group.

		All Tumors (*n* = 28)		BRCA1 (*n* = 13)		BRCA2 (*n* = 15)	
		Median (10th, 90th Percentile)		Median (10th, 90th Percentile)		Median (10th, 90th Percentile)	
Age at Diagnosis		47 (32, 61)		47 (30, 51)		46 (33, 62)	
Time From BRCA Diagnosis to Cancer Diagnosis (Months)		59.3 (14.9, 116.4)		63.2 (6.7, 144.9)		44.4 (23.1, 102.4)	
		***n***	**(%)**	***n***	**(%)**	***n***	**(%)**
DCIS		4	14	1	7	3	20
IDC		24	86	12	93	12	80
ER status	Negative	9	32	8	62	1	7
	Positive	18	64	4	31	14	93
	Unknown	1	4	1	7	0	0
PR status	Negative	13	46	9	69	4	27
	Positive	11	39	3	23	8	53
	Unknown	4	15	1	8	3	20
Her2 status	Negative	18	64	8	62	10	67
	Positive	5	18	4	31	1	7
	Unknown	5	18	1	8	4	27
Triple negative	Yes	7	25	6	46	1	7
Tumor size (mm)	<20	24	89	11	85	13	87
	>21	3	10	2	15	1	6.5
	Unknown	1		0	0	1	6.5
Node positive	Yes	5	18	2	15	3	20
	Unknown	2	7	0	0	2	13
Compliant	Yes	23	79	10	77	13	87

DCIS: ductal carcinoma in situ; IDC: invasive ductal carcinoma; ER: estrogen receptor; PR: progesterone receptor.

**Table 3:** Cancer Characteristics: PM/IBR.

		All Tumors (*n* = 8)	BRCA1 (*n* = 4)	BRCA2 (*n* = 4)
		Median (10th, 90th percentile)	Median (10th, 90th percentile)	Median (10th, 90th percentile)
Age at Diagnosis		41 (34, 63)	39 (34, 48)	51 (34, 63)
Time from BRCA Diagnosis to Cancer Diagnosis (months)		32.0 (2.7, 87.5)	47.2 (16.9, 87.5)	26.9 (2.7, 49.6)
		***n***	***n***	***n***
DCIS		2	2	0
IDC		6	2	4
ER status	Negative	4	2	2
	Positive	4	2	2
PR status	Negative	6	4	2
	Positive	2	0	2
Her2 status	Negative	4	2	2
	Positive	3	1	2
	Unknown	1	1	0
Triple negative	Yes	2	2	0
Tumor size (mm)	<20	6	3	3
	>21	2	1	1
Node positive	Yes	2	0	2
	Unknown	3	2	1
Compliant	Yes	7	4	3

DCIS: ductal carcinoma in situ; IDC: invasive ductal carcinoma; ER: estrogen receptor; PR: progesterone receptor.

**Table 4:** Time Periods Leading to Surgery.

T1 (BRCA Mutation Diagnosis to Decision for Surgery)	Frequency (*n*)	Percent (%)
HCP visit and referral for PM/IBR </= 3 months from BRCA mutation disclosure	41	39.1
Initially declined surgical consult then changed mind and proceeded for unknown reason	24	22.9
Initially declined surgical consult then changed mind due to family influence (i.e., new cancer)	9	8.6
Delayed in order to proceed with BSO first	7	6.7
Initially declined surgical consult then changed mind after completing child-bearing or breast feeding	5	4.8
Delayed due to patient distance from tertiary care center	5	4.8
Initially declined surgical consult then changed mind after imaging abnormality	4	3.8
Initially declined surgical consult then changed mind due to new information (discussion with new physician, media influence)	2	1.9
Interested in PM/IBR but deemed unable to have reconstruction	3	2.9
Delay for unknown reason	3	2.9
Delay for patient health issues	2	1.9
**T2 (HCP Referral to Consultation with Surgeon)**	**Frequency (*n*)**	**Percent (%)**
HCP referral to consultation with surgeon </= 6 months	64	70.3
Change in requested surgeon	8	8.8
Long wait time for consultation	7	7.7
Unknown	7	7.7
Patient cancelled or postponed	4	4.4
Delay for patient health issues	1	1.1
**T3 (Surgical Consultation to Surgery)**	**Frequency (*n*)**	**Percent (%)**
Surgery </= 1 year from surgical consult	58	61.7
Patient undecided after consult or patient timing preference	12	12.8
Patient requested second opinion	8	8.5
Long surgical wait list	7	7.5
Patient health (BMI, smoking, nursing)	5	5.3
Unknown	2	2.1
Patient moved before surgery completed	1	1.1
Patient elected to have BSO completed first	1	1.1

BSO: bilateral salpingo-oophorectomy; BMI: body mass index; HCP: hereditary cancer program.

**Table 5:** Cancer Characteristics: PM/IBR.

Patient	Interval from BRCA Diagnosis to Cancer Diagnosis (Months)	Age at Cancer Diagnosis	Tumor Size (mm)	No. Positive Nodes/ Nodes Assessed	Histologic Type	Grade	ER/PR Status	Her2 Status	Findings CBE	Findings MRI	Findings MMG	BRCA	Compliance
1 *	2	63	<1	N/A	IDC	2	-/-	+	N	N/A	N/A	2	Y
2 *	7	35	20	0/2	IDC	2	+/+	+	N/A	N/A	N	2	Y
3	16	39	5	0/4	DCIS	3	+/-	+	N	Atypical	N	1	Y
4	18	43	70	N/A	DCIS	2	+/-	N/A	N	Suspicious	Biopsy recommended	1	Y
5	45	65	29	5/17	IDC	2	-/-	-	N	N/A	N/A	2	Y
6	49	44	10	7/12	IDC	2	+/+	-	Mass detected	N/A	Indeterminate	2	N
7 *	76	55	2	N/A	IDC	3	-/-	-	N	N	N	1	Y
8	85	41	9	0/5	IDC	3	-/-	-	Mass detected	Malignant mass	Malignant mass	1	Y

IDC: invasive ductal carcinoma; DCIS: ductal carcinoma in situ; N: normal; N/A: not available; CBE: clinical breast examination; MRI: magnetic resonance imaging; MMG: mammogram. Compliance was defined as imaging done at a frequency of q12 months or less. * Diagnosed at surgery.

## Data Availability

Restrictions apply to the availability of these data. Data was obtained from the British Columbia Cancer Agency (BCCA) and shared through a formal BCCA Health Research Information Sharing Agreement. Data are unavailable unless accessed on the premises of the BCCA with the permission of the BCCA.

## References

[1] Walsh C.S. (2015). Two decades beyond BRCA1/2: Homologous recombination, hereditary cancer risk and a target for ovarian cancer therapy. Gynecol. Oncol..

[2] https://seer.cancer.gov/statfacts/html/breast.html.

[3] King M.C., Marks J.H., Mandell J.B. (2003). Breast and ovarian cancer risks due to inherited mutations in BRCA1 and BRCA2. Science.

[4] Burke W., Daly M., Garber J., Botkin J., Kahn M.J., Lynch P., McTiernan A., Offit K., Perlman J., Petersen G. (1997). Recommendations for follow-up care of individuals with an inherited predisposition to cancer. II. BRCA1 and BRCA2. Cancer Genetics Studies Consortium. JAMA.

[5] Sakorafas G.H. (2003). The management of women at high risk for the development of breast cancer: Risk estimation and preventative strategies. Cancer Treat. Rev..

[6] Stoutjesdijk M.J., Boetes C., Jager G.J., Beex L., Bult P., Hendriks J.C.L., Laheij R.J.F., Massuger L., Van Die L.E., Barentsz J.O. (2001). Magnetic resonance imaging and mammography in women with a hereditary risk of breast cancer. J. Natl. Cancer Inst..

[7] Meijers-Heijboer E.J., Verhoog L.C., Brekelmans C.T., Seynaeve C., Tilanus-Linthorst M.M., Wagner A., Dukel L., Devilee P., Van Den Ouweland A.M., Klijn J.G. (2000). Presymptomatic DNA testing and prophylactic surgery in families with a BRCA1 or BRCA2 mutation. Lancet.

[8] Heemskerk-Gerritsen B.A., Menke-Pluijmers M.B., Jager A., Tilanus-Linthorst M.M.A., Koppert L.B., Obdeijn I.M.A., Van Deurzen C.H.M., Collée J.M., Seynaeve C., Hooning M.J. (2013). Substantial breast cancer risk reduction and potential survival benefit after bilateral mastectomy when compared with surveillance in healthy BRCA1 and BRCA2 mutation carriers: A prospective analysis. Ann. Oncol..

[9] Pijpe A., Andrieu N., Easton D.F., Kesminiene A., Cardis E., Noguès C., Gauthier-Villars M., Lasset C., Fricker J., Peock S. (2012). Exposure to diagnostic radiation and risk of breast cancer among carriers of BRCA1/2 mutations: Retrospective cohort study (GENE-RAD-RISK). BMJ.

[10] Lodder L.N., Frets P.G., Trijsburg R.W., Meijers-Heijboer E.J., Klijn j.M., Seynaeve C., van Geel A.N., Tilanus M.M.A., Bartels C.C.M., Verhoog L.C. (2002). One year follow-up of women opting for presymptomatic testing for BRCA1 and BRCA2: Emotional impact of the test outcome and decisions on risk management (surveillance or prophylactic surgery). Breast Cancer Res. Treat..

[11] Howard A.F., Balneaves L.G., Bottorff J.L., Rodney P. (2011). Preserving the self: The process of decision making about hereditary breast cancer and ovarian cancer risk reduction. Qual Health Res..

[12] National Institute for Health and Care Excellence (NICE) (2013). NICE Clinical Guideline 164 Familial Breast Cancer: Classification and Care of People at Risk of Familial Breast Cancer and Management of Breast Cancer and Related Risks in People with a Family History of Breast Cancer. https://www.nice.org.uk/guidance/cg164.

[13] Meijers-Heijboer H., Van Geel B., Van Putten W.L.J., Henzen-Logmans S.C., Seynaeve C., Menke-Pluymers M.B., Bartels C.C., Verhoog L.C., Van Den Ouweland A.M., Niermeijer M.F. (2001). Breast cancer after prophylactic bilateral mastectomy in women with a BRCA1 or BRCA2 mutation. N. Engl. J. Med..

[14] Rebbick T.R., Friebel T.M., Lynch H.T., Neuhausen S.L., Veer L.v., Garber J.E., Evans G.R., Narod S.A., Isaacs C., Matloff E. (2004). Bilateral Prophylactic Mastectomy Reduces Breast Cancer Risk in *BRCA1* and *BRCA2* Mutation Carriers: The PROSE Study Group. J. Clin. Oncol..

[15] Domchek S.M., Friebel T.M., Singer C.F., Evans D.G., Lynch H.T., Isaacs C., Garber J.E., Neuhausen S.L., Matloff E., Eeles R. (2010). Association of risk-reducing surgery in BRCA1 or BRCA2 mutation carriers with cancer risk and mortality. JAMA.

[16] Skytte A.B., Gerdes A.M., Andersen M.K., Sunde L., Brøndum-Nielsen K., Waldstrøm M., Kølvraa S., Crüger D. (2010). Risk-reducing mastectomy and salpingo-oophorectomy in unaffected BRCA mutation carriers: Uptake and timing. Clin. Genet..

[17] Ingham S.L., Sperrin M., Baildam A., Ross G.L., Clayton R., Lalloo F., Buchan I., Howell A., Evans D.G.R. (2013). Risk-reducing surgery increases survival in BRCA1/2 mutation carriers unaffected at time of family referral. Breast Cancer Res. Treat..

[18] Kaas R., Verhoef S., Wesseling J., Rookus M.A., Oldenburg H.S.A., Peeters M.V., Rutgers E.J.T. (2010). Prophylactic mastectomy in BRCA1 and BRCA2 mutation carriers: Very low risk for subsequent breast cancer. Ann. Surg..

[19] Ludwig K.K., Neuner J., Butler A., Geurts J.L., Kong A.L. (2016). Risk reduction and survival benefit of prophylactic surgery in BRCA mutation carriers, A systematic review. Am. J. Surg..

[20] Li X., You R., Wang X., Liu C., Xu Z., Zhou J., Yu B., Xu T., Cai H., Zou Q. (2016). Effectiveness of Prophylactic Surgeries in BRCA1 or BRCA2 Mutation Carriers: A Meta-analysis and Systematic Review. Clin. Cancer Res..

[21] Yao K., Liederback E., Tang R., Lei L., Czechura T., Sisco M., Howard M., Hulick P.J., Weissman S., Winchester D.J. (2015). Nipple-sparing mastectomy in BRCA1 and BRCA2 mutation carriers: An interim analysis and review of the literature. Ann. Surg. Oncol..

[22] Beattie M.S., Crawford B., Lin F., Ziegler E.V.J. (2009). Uptake, time course, and predictors of risk-reducing surgeries in BRCA carriers. Genet. Test. Mol. Biomark..

[23] Flippo-Morton T., Walsh K., Chambers K., Amacker-North L., White B., Sarantou T., Boselli D.M., White R.L. (2016). Surgical decision making in the BRCA-positive population: Institutional experience and comparison with recent literature. Breast.

[24] Lerman C., Narod S., Schulman K., Chanita Hughes M.S., Andres Gomez-Caminero M.P.H., George Bonney P., Karen Gold P., Bruce Trock P., David Main M.S. (1996). BRCA1 testing in families with hereditary breast-ovarian cancer. A prospective study of patient decision-making and outcomes. JAMA.

[25] Metcalfe K.A., Liede A., Hoodfar E., Scott A., Foulkes W.D., Narod S.A. (2000). An evaluation of needs of female BRCA1 and BRCA2 carriers undergoing genetic counseling. J. Med. Genet..

[26] Metcalfe K.A., Dennis C.L., Poll A., Armel S., Demsky R., Carlsson L., Nanda S., Kiss A., Narod S.A. (2017). Effect of decision aid for breast cancer prevention on decisional conflict in women with a *BRCA1* or *BRCA2* mutation: A multisite, randomized, controlled trial. Gen. Med..

[27] Schwartz M.D., Valdimarsdottir H.B., DeMarco T.A., Peshkin B.N., Lawrence W., Rispoli J., Brown K., Isaacs C., O’Neill S., Shelby R. (2009). Randomized trial of a decision aid for BRCA1/BRCA2 mutation carriers: Impact on measures of decision making and satisfaction. Health Psychol..

[28] Siciliani L., Hurst J. (2003). Explaining Waiting Times Variations for Elective Surgery Across OECD Countries.

